# A Method to Improve the Imaging Quality in Dual-Wavelength Digital Holographic Microscopy

**DOI:** 10.1155/2018/4582590

**Published:** 2018-10-14

**Authors:** Yanan Zeng, Junsheng Lu, Xinyu Chang, Yuan Liu, Xiaodong Hu, Kangyan Su, Xiayu Chen

**Affiliations:** ^1^College of Engineering and Technology, Tianjin Agricultural University, Tianjin 300000, China; ^2^State Key Laboratory of Precision Measuring Technology and Instruments, Tianjin University, 300072 Tianjin, China

## Abstract

A digital hologram-optimizing method was proposed to improve the imaging quality of dual-wavelength digital holographic microscopy (DDHM) by reducing the phase noise level. In our previous work, phase noise reduction was achieved by dual-wavelength digital image-plane holographic microscopy (DDIPHM). In the optimization method in this paper, the phase noise was further reduced by enhancing the real-image term and suppressing effects of the zero-order term in the frequency spectrum of a digital hologram. Practically, the carrier frequency of the real-image term has the correspondence with interference fringes in the hologram. Mathematically, the first order intrinsic mode function (IMF1) in bidimensional empirical mode decomposition (BEMD) has similar characteristics to the grayscale values of ideal interference fringes. Therefore, with the combination of DDIPHM and BEMD, by utilizing the characteristics of IMF1, the digital hologram was optimized with purified interference fringes, enhancing the real-image term simultaneously. Finally, the validity of the proposed method was verified by experimental results on a microstep.

## 1. Introduction

With various advantages such as the real-time performance, noninvasive property, and easy processing by mathematical computing, digital holographic microscopy (DHM) has experienced substantial development in surface profile measurement of microstructures [1–3]. The object wavefront can be retrieved in amplitude and phase by the numerical reconstruction process of a digital hologram simultaneously [4]. Dual-wavelength digital holographic microscopy (DDHM) extends the measurement range of single-wavelength digital holographic microscopy when DHM is applied to measure high aspect-ratio structures, especially the step structures with the micron step height [5, 6].

However, the phase noise, especially in the recording process, is amplified when the measurement height range is amplified simultaneously, resulting in a loss of axial resolution in the measurement [7, 8].

Except for image processing methods [9–11], several noise-reducing approaches aimed at DDHM have been proposed in the last decades, such as the mathematic methods, the dual-wavelength unwrapping algorithms [7, 8]. Error points occur when the dual-wavelength unwrapping algorithms are applied. In previous work, we analyzed the reasons for occurrence of error points and proposed a much safer method, namely, dual-wavelength digital image-plane holographic microscopy (DDIPHM) [12, 13] to suppress the phase noise in DDHM. In this paper, an optimization method based on combination of bidimensional empirical mode decomposition (BEMD) and DDIPHM was put forward to improve the imaging quality of DDHM.

The empirical mode decomposition (EMD) method has been used in digital holography. EMD directly performs the task of particle sizing and axial locating from in-line digital holograms rather than reconstructing the optical field [14, 15]. As for noise reduction, EMD is utilized as a universal data filter. The reconstructed intensity images are decomposed by EMD. Removing the intrinsic mode functions from reconstructed intensity images, the remaining terms are the denoised images [16]. The EMD method is applied at the last step in image processing. EMD plays the smoothing role in noise reduction. Therefore, the noises are not actually analyzed.

In this paper, different from [16], BEMD was used on the original digital hologram to analyze and process the frequency spectrum. The optimization method proposed in this paper combined BEMD and DDIPHM. After applying the proposed method, the interference fringes of holograms were purified and enhanced. As a result, the zero-order term in the frequency spectrum was suppressed. Therefore, reconstructed phase noise was reduced in comparison to DDIPHM. By optimizing the hologram from interference fringes, the imaging quality could be improved from the bottom. The digital image-plane microscopic hologram of a microstep was processed as the sample to verify the method proposed in DDHM.

## 2. Experimental Apparatus

**Figure 1:**
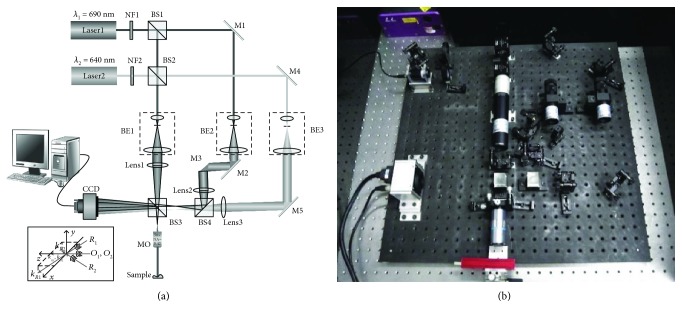
Experimental setup for DDHM. (a) The illustration of the DDHM system. (b) The apparatus of DDHM. NF1 and NF2: variable neutral filters; BS1–BS4: beam splitters; M1–M5: mirrors; BE1–BE3: beam expanders; MO: microscope objective with magnification 50x and numerical aperture NA = 0.42; Lens1–Lens3: lens. Inset: 3D distribution of the incident wave propagation directions upon the CCD plane; *k*_R1_ and *k*_R2_ are the propagation direction vectors of the reference waves *R*_1_ for wavelength *λ*_1_ and *R*_2_ for *λ*_2_. *k*_O1_ and *k*_O2_ are the vectors of object waves.

The experimental setup for DDHM is depicted in Figure 1. The illumination sources included a tunable diode laser at *λ*_1_ = 690 nm (Nanobase, Xperay-TL-STD, 639 nm–697 nm) and a diode-pumped laser at *λ*_2_ = 640 nm (CrystaLaser, CL640-050-S), yielding the beat-wavelength Λ = 8.832 *μ*m. The neutral filters NF1 and NF2 were used to adjust the intensities of two laser beams. After passing through the beam splitters BS1 and BS2, the two laser beams were split into the object beam and reference beam, respectively. The information of the sample collected by a microscope objective (MO, Mitutoyo, M Plan Apo SL NA = 0.42, 50x) was coded in an interference pattern from the object beam and the reference beam. This interference pattern was recorded on the digital detector (CCD, Imperx, PX-2M30-L, *M* × *N* = 1008 × 1028, square pixel view of 7.4 *μ*m, 33 frames/s) to form the hologram. The hologram is special as it is the focused image of the tested sample, namely, the image-plane hologram. All of the beams were collimated and expanded by the beam expanders BE1, BE2, and BE3. The lenses in Figure 1 were used to produce spherical waves. By tilting mirrors M3 and M5, the k-vectors of each wavelength can be tuned independently. Afterwards, the orientation and quantity of fringes were tuned with orthogonal carrier frequencies to avoid the overlapping effect in the frequency spectrum.

## 3. Principle

The imaging noise of DDHM originates from coherent recording and the finite size of the pixels in the CCD camera. The temperature variation in media and visible blemishes on any window where light passes through can also cause diffraction and reflection. The above-mentioned disturbing factors should be removed at the stage of hologram processing; otherwise, they would introduce phase noises in the measurement for surface profiling of microstructures.

The intensity of the digital hologram recorded in single-wavelength DHM can be written as

(1) $$\begin{array}{c} I\left(x,y\right)=O{\left(x,y\right)}^{2}+R{\left(x,y\right)}^{2}+O^{*}R\left(x,y\right)+{OR}^{*}\left(x,y\right). \end{array}$$

*O*(*x*, *y*) was the object wavefront, while *R*(*x*, *y*) was the reference wavefront. ^∗^ denoted the conjugative term. In (1), the real-image term *O*^∗^*R*(*x*, *y*) should be extracted by filtering the frequency spectrum of the recorded hologram to retrieve the phase. The disturbing factors mentioned in the recording process, including high frequency factors, such as speckle noises, and low frequency factors, such as uniform illumination, were located in the full frequency spectrum. Hence, the filtered *O*^∗^*R*(*x*, *y*) would be affected, resulting in phase noises.

However, the carrier frequency of *O*^∗^*R*(*x*, *y*) and *OR*^∗^(*x*, *y*) had the correspondence with the intensive interference fringes. In fact, the interference fringes in the space domain corresponded to the carrier frequency of *O*^∗^*R*(*x*, *y*) and *OR*^∗^(*x*, *y*) in the frequency domain. Thus, extracting interference fringes from a hologram would suppress the zero-order term and enhance the real-image term in the frequency spectrum. Therefore, with the enhanced *O*^∗^*R*(*x*, *y*), phase noises would be reduced. The EMD method happened to solve this problem.

EMD decomposes a complex time series into the sum of a limited number of IMFs. Each IMF needs to satisfy the following two conditions:

1. The number of extreme points should be equal to or larger than the number of zero points in the entire time series
2. At any point, the mean value of the envelopes formed by the local maximum point and the local minimum point is zero

**Figure 2:**
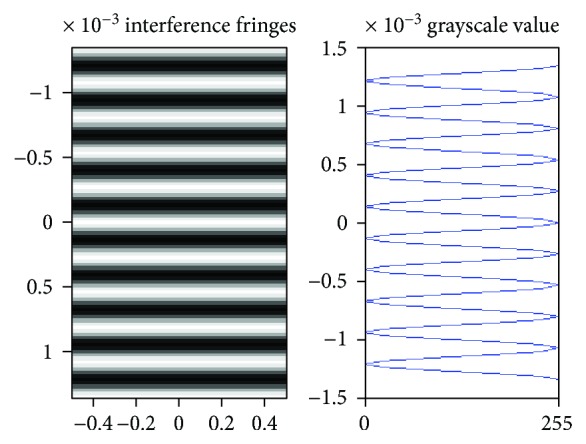
The interference fringes in grayscale value of Young's double-slit interference.

Figure 2 shows the analog interference fringes and grayscale value of Young's double-slit interference (wavelength *λ* = 636 nm, width of slits *d* = 0.002 m, distance between the recording plane and slits *D* = 1 m). The characteristics of IMF are well matched to the grayscale value of interference fringes in the hologram.

Therefore, the information of interference fringes can be obtained by the sifting process of the hologram. Since the hologram is two-dimensional, the BEMD sifting process is applied and described as follows [14, 15].

1. *h*_*ij*_ = *s*, *h*_*ij*_: process variable; *i* and *j*: cycle time; *s*: initial signal; *i* = 1, *j* = 1
2. Identify sets of minima (*A*) and maxima (*B*) of *h*_*ij*_. If there are none, save *h*_*ij*_ as a residue *r*_*i*_ and finish the algorithm
3. Connect all the local maxima of *h*_*ij*_ to create the upper envelope, and similarly for the lower envelope of *h*_*ij*_, calculate the arithmetic mean value *E*_*ij*_
4. *H* = *h*_*ij*_, *T*_*ij*_ = *h*_*ij*_ − *E*_*ij*_
5. If the subtraction result meets the IMF condition, save *T*_*ij*_ as an IMF*i*, *i* = *i* + 1 and go back to step 2 with *h*_*ij*_ = *H* − *T*_*ij*_. Otherwise, *j* = *j* + 1, *h*_*ij*_ = *T*_*ij*_ and go back to step 2

By analyzing the frequency spectrum of the hologram, the hologram is decomposed by BEMD, and IMF1 can be remained as the optimized hologram with the processed frequency spectrum to be calculated in the reconstruction.

The intensity distribution of the hologram of DDHM can be written as

(2) $$\begin{array}{c} I_{IPH}\left(x,y\right)=\overset{2}{\underset{i=1}{\sum }}\left({\left|R_{i}\right|}^{2}+{\left|O_{i}\right|}^{2}+R_{i}{O_{i}}^{*}+{R_{i}}^{*}O_{i}\right). \end{array}$$

**Figure 3:**
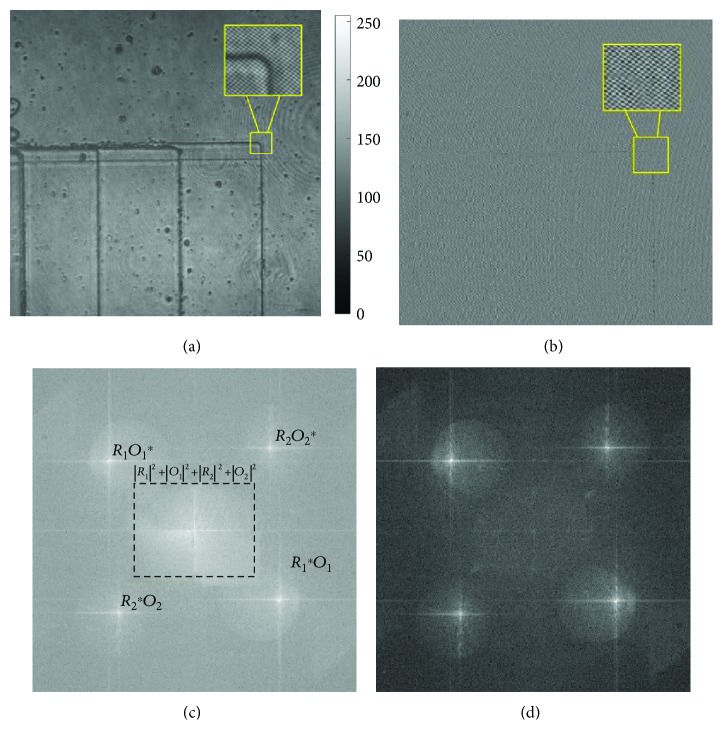
Experimental hologram and frequency spectrum. (a) Image-plane hologram. (b) IMF1 of the image-plane hologram. (c) Frequency spectrum of the hologram. (d) Frequency spectrum of IMF1. The boxes in figures are the magnified parts.

*I*_IPH_ is the intensity of an image-plane hologram. (*x*, *y*) is the coordinate of the image-plane hologram, *i* = 1, 2. *O*_*i*_(*i* = 1, 2) is the complex amplitude of the object beam of each wavelength. *R*_*i*_(*i* = 1, 2) is the complex amplitude of the reference beam. ^∗^ denotes the complex conjugate term. Due to different angles of k-vectors, each term in (2) occupies a different position in the Fourier plane without overlap, as seen in Figure 3(c). With the method of BEMD, the IMF1 term of the original hologram is regarded as the optimized hologram (Figure 3(b)). The intensity is labeled as *I*_IMF1_ in Figure 3(b). The frequency spectrum is shown in Figure 3(d). According to Figure 3(c), the frequency component of the real image *R*_*i*_^∗^*O*_*i*_ or the virtual image *R*_*i*_^∗^*O*_*i*_ is filtered:

(3) $$\begin{array}{c} R_{i}\left(x,y\right){O_{i}}^{*}\left(x,y\right)=IFT\left\{W_{i}\left(\xi ,\eta \right)\text{FT}\left[I_{IMF1}\left(x,y\right)\right]\right\}, \end{array}$$

where FT and IFT denote the Fourier transform and inverse Fourier transform, respectively. *W*_*i*_(*ξ*, *η*) is the window function for frequency filtering.

By using DDIPHM, the phase and amplitude of the sample can be straightforwardly extracted:

(4) $$\begin{array}{c} u_{i}\left(x,y\right)=\Gamma_{i}\left(x,y\right)\left[R_{i}\left(x,y\right){O_{i}}^{*}\left(x,y\right)\right], \end{array}$$

where *u*_*i*_(*x*, *y*) is the reconstructed wavefront for wavelength *λ*_1_. Γ_*i*_(*x*, *y*) is the digital phase mask to compensate for aberrations. The phase of *λ*_*i*_ is

(5) ϕix,y=arctanImuix,yReuix,y.

The height of the sample is

(6) $$\begin{array}{c} h\left(x,y\right)=\frac{\phi_{1}\left(x,y\right)-\phi_{2}\left(x,y\right)}{4\pi }\times \frac{\lambda_{1}\lambda_{2}}{\left|\lambda_{2}-\lambda_{1}\right|}=\frac{\Phi \left(x,y\right)\Lambda }{4\pi }, \end{array}$$

where Φ(*x*, *y*) is the synthetized phase and Λ is beat-wavelength, Λ = *λ*_1_*λ*_2_/*λ*_1_ − *λ*_2_.

## 4. Experimental Results

The experimental results should be discussed from the perspectives of previous studies and working hypotheses. The findings and their implications should be discussed in the broadest context. Future research directions may also be highlighted.

To assess the validity, a microstep (surface gold-plated, a testing sample of Lyncee tec) was measured by the setup of DDHM, and a stylus profilometer (KLA-Tencor, P-16+/P-6) with the force of 1 mg for comparison. In this part, the experimental results of DDIPHM, DDIPHM with BEMD, and DDHM are compared to demonstrate that BEMD can achieve a lower phase noise level.

The image-plane hologram of the microstep is presented in Figure 3(a). The magnified part of interference fringes shows the spatial frequency of the two wavelengths with different angles. Figure 3(b) is the IMF1 of Figure 3(a) after using the BEMD method. In Figure 3(b), the interference fringes stand out from the background. The frequency spectrums of Figures 3(a) and 3(b) are shown in Figures 3(c) and 3(d), respectively. The separated terms of (1) are labeled in Figure 3(c). After BEMD processing, ∑_*i*=1_^2^(|*R*_*i*_|^2^ + |*O*_*i*_|^2^) is reduced. Actually, other disturbing factors with high or low frequency are also reduced as the interference terms are enhanced in IMF1. The separated terms in the frequency spectrum in Figures 3(c) and 3(d) demonstrate that each frequency component can be straightforwardly isolated by spatial filtering.

**Figure 4:**
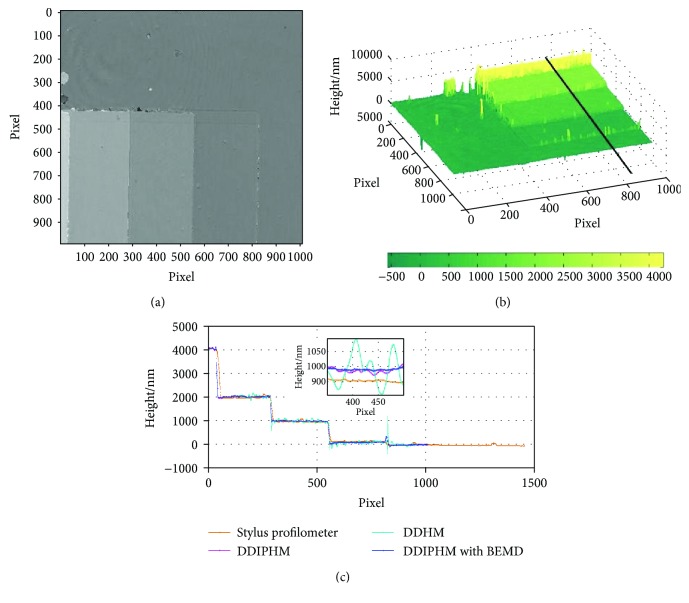
The experimental results of DDIPHM with BEMD. (a) The phase image of microstep measured by DDIPHM with BEMD. (b) The surface profile of the microstep measured by DDIPHM with BEMD. (c) The height profile lines plotted along the black line in Figure 4(b) measured through stylus profilometry, DDIPHM, DDIPHM with BEMD, and DDHM (angular spectrum method, reconstruction distance *d* = 75 mm). The black rectangular shows the magnified part.

Figure 4(a) shows the reconstructed phases. The surface profile of the microstep is shown in Figure 4(b).  Figure 4(c) demonstrates the height profile plotted along the black line through stylus profilometry, DDIPHM, DDIPHM with BEMD, and DDHM in Figure 4(b) (reconstruction distance is *d* = 75 mm, reconstructed by the angular spectrum method).

Since the precision of DHM can be 0.1 nm, the calculated height value was kept one decimal digit. The average height of multiple profile lines is the experimental results (Figure 4(c) and Table 1) of DDIPHM, DDIPHM with BEMD, stylus profilometry, and DHM after removing the gross error like apparent stains. The steps are numbered as 1, 2, 3, and 4 from left to right.

## 5. Discussion

Two points can be concluded from the experimental results: first, compared to DDIPHM, the noise is obviously suppressed in the measurement of DDIPHM with BEMD; second, compared to stylus profilometry, the measuring correctness of DDIPHM with BEMD is verified by the good accordance of the two measurement results. Since BEDM is used to enhance the contrast of interference fringes, DDIPHM with BEMD is especially suitable for the reconstruction of holograms acquired in the environment with speckle noises. The refractive index difference between biological cells or tissues and environment can be quite large. Therefore, DDIPHM with BEMD was meant to be the appropriate method to retrieve the phase of biological samples. Though the phase range of the measurement was enlarged, the lateral resolution was maintained.

## 6. Conclusions

In this paper, a hologram-optimizing method was proposed. By using the DDIPHM with BEMD method, the interference fringes were extracted, resulting in the enhancement of the real-image term and suppression of the zero-order term in the frequency spectrum of the hologram. The affection of disturbing factors in the recording process was suppressed simultaneously. According to the experimental results, the measured noise level of the DDIPHM with BEMD method can be further reduced compared to DDIPHM. The validity of the proposed method was verified compared to stylus profilometer measurement.

## Figures and Tables

**Table 1:** The microstep height experimental results.

Step number	1	2	3	4
DDIPHM	4048.1 ± 18.8 nm	2021.1 ± 16.8 nm	988.5 ± 15.9 nm	89.4 ± 20.6 nm
DDIPHM with BEMD	4043.1 ± 12.1 nm	2027.1 ± 10.2 nm	986.5 ± 9.3 nm	83.5 ± 10.3 nm
Stylus profilometer	4030.8 ± 17.2 nm	1980.0 ± 13.5 nm	963.7 ± 10.7 nm	130.0 ± 9.1 nm
Classical DDHM	—	2032.4 ± 54.8 nm	994.3 ± 52.5 nm	89.6 ± 42.5 nm

## Data Availability

The data used to support the findings of this study are included within the article.
